# Minocycline impairs TNF-α-induced cell fusion of M13SV1-Cre cells with MDA-MB-435-pFDR1 cells by suppressing NF-κB transcriptional activity and its induction of target-gene expression of fusion-relevant factors

**DOI:** 10.1186/s12964-019-0384-9

**Published:** 2019-07-02

**Authors:** Julian Weiler, Thomas Dittmar

**Affiliations:** Institute of Immunology, Centre of Biomedical Education and Research (ZBAF), Witten/Herdecke University, Stockumer Str. 10, 58448 Witten, Germany

**Keywords:** Minocycline, Cell fusion, TNF-α, NF-κB, Breast cancer

## Abstract

**Background:**

To date, several studies have confirmed that driving forces of the inflammatory tumour microenvironment trigger spontaneous cancer cell fusion. However, less is known about the underlying factors and mechanisms that facilitate inflammation-induced cell fusion of a cancer cell with a normal cell. Recently, we demonstrated that minocycline, a tetracycline antibiotic, successfully inhibited the TNF-α-induced fusion of MDA-MB-435 cancer cells with M13SV1 breast epithelial cells. Here, we investigated how minocycline interferes with the TNF-α induced signal transduction pathway.

**Methods:**

A Cre-LoxP recombination system was used to quantify the fusion of MDA-MB-435-pFDR1 cancer cells and M13SV1-Cre breast epithelial cells. The impact of minocycline on the TNF-α signalling pathway was determined by western blotting. The transcriptional activity of NF-κB was characterised by immunocytochemistry, western blot and ChIP analyses. An NF-κB-luciferase reporter assay was indicative of NF-κB activity.

**Results:**

Minocycline treatment successfully inhibited the TNFR1-TRAF2 interaction in both cell types, while minocycline abrogated the phosphorylation of IκBα and NF-κB-p65 to suppress nuclear NF-κB and its promotor activity only in M13SV1-Cre cells, which attenuated the expression of MMP9 and ICAM1. In MDA-MB-435-pFDR1 cells, minocycline increased the activity of NF-κB, leading to greater nuclear accumulation of NF-κB-p65, thus increasing promoter activity to stimulate the expression of ICAM1. Even though TNF-α also activated all MAPKs (ERK1/2, p38 and JNK), minocycline differentially affected these kinases to either inhibit or stimulate their activation. Moreover, SRC activation was analysed as an upstream activator of MAPKs, but no activation by TNF-α was revealed. The addition of several specific inhibitors that block the activation of SRC, MAPKs, AP-1 and NF-κB confirmed that only NF-κB inhibition was successful in inhibiting the TNF-α-induced cell fusion process.

**Conclusion:**

Minocycline is a potent inhibitor in the TNF-α-induced cell fusion process by targeting the NF-κB pathway. Thus, minocycline prevented NF-κB activation and nuclear translocation to abolish the target-gene expression of MMP9 and ICAM1 in M13SV1-Cre cells, resulting in reduced cell fusion frequency.

## Background

The process of cell fusion is a common widespread biological phenomenon and is involved in numerous physiological events throughout the body [1–3]. Accordingly, merging two or more cells induces the determination and differentiation of certain novel cell types, such as those from myoblast fusion [4] and osteoclast maturation [5], or the formation and development of a new organ complex, for instance, during placentation [6]. However, in some cases, cell fusion can cause diverse pathophysiological disorders such as those from virus-cell fusion or can force tumorigenesis as a consequence of spontaneous cell–cell fusion [1]. In addition, several studies in vitro and in vivo have reported that cell fusion gives rise to tumour cell hybrids with a high malignancy potential, as has been observed in various cancer types [7–9]. Even though the role of cell fusion in tumorigenesis has already been detected by several studies, the underlying mechanism that drives this fusion process is largely unknown. Despite the diversity of the cell types that undergo cell fusion in multicellular organisms, the process is the same [2]. Cell fusion is a multistep process that can be subdivided into priming, chemotaxis, adhesion, fusion and postfusion phases, which include adhesion molecules, intracellular signal proteins, proteases, transcription factors and cell-organising proteins [2]. To date, few fusion proteins are known to be involved in the cell fusion process and include, for example, syncytin-1 and syncytin-2, which are necessary for trophoblast fusion, and the tetraspanin protein CD-9, which is required to initiate sperm–egg fusion. Likewise, macrophage fusion depends on the expression of several fusion markers, such as E-cadherin, CD-47 or RAC1 [6, 10, 11]. Moreover, several soluble factors are known to participate in the cell fusion process, including those ranging from chemokines, such as CCL-2 [11] and CXCL12 [12], to matrix-metalloproteases (MMPs), such as MMP9, ADAM10, ADAM12 or MT1-MMP [13–16]. How these effectors can contribute to cell fusion differs; for instance, chemokines, such as CCL-2, might be important for the chemotaxis of diverse cell types within the body by recruiting them towards their fusion partners [11], while metalloproteinases might be capable of advancing the merge of plasma membranes by cutting off cell-surface receptors to reduce the distance between cells and thus facilitating cell merging [14, 17]. In addition, several cytokines, such as IL-4, IL-13, RANKL, and TNF-α, seem to play an important role in the process of macrophage fusion [11], myoblast fusion [18] and even in tumour-hybrid formation [9, 13]. Specifically, IL-4 is a crucial cytokine for myoblast fusion as it leads the migration of myoblasts towards growing multinucleated muscle cells by activating the NFATC2 pathway and thus promoting fusion [18]. For macrophage fusion, cytokines such as IL-4, IL-13, M-CSF and RANKL act as molecular mediators by triggering the macrophage fusion that results in a multinucleated giant cell through the activation of transcription factor STAT6 [11]. Likewise, cytokines such as IL-1β, IL-4, IL-13 and TNF-α facilitate the cell fusion process that results in increased tumour-hybrid formation [9, 19, 20]. It is thought that cytokines regulate the cell fusion process through activation of distinct signalling pathways and transcription factors, thereby transferring cells into a fusion-competent status. In this regard, inflammation might be a potent inducer for cell fusion due to the release of many cytokines accompanied by diverse migrating immunocompetent cells and stroma cells to the side of the injury to create a fusion-friendly milieu. Over the last decade, increasing evidence has indicated that the cell fusion frequency in several kinds of cancers was enhanced by the pro-inflammatory cytokine TNF-α [9, 20, 21]. Nevertheless, the signalling mechanisms by which TNF-α manages this fusion process remain poorly understood. Yan et al. recently showed that cell fusion between oral cancer cells and vascular endothelial cells could be enhanced by TNF-α via a Wnt/β-catenin-dependent pathway, resulting in the upregulation of syncytin-1/ASCT2 and the promotion of cell fusion [21]. In addition, we demonstrated that the cell fusion frequency of MDA-MB-435 cancer cells with M13SV1 breast epithelial cells was markedly increased by TNF-α, which in turn could be blocked by the administration of the antibiotic minocycline [13]. Minocycline, a second-generation derivative of tetracycline, exerts widespread beneficial effects with antimicrobial, anti-inflammatory, neuroprotective and even anti-tumorigenic protection inside the body [22–25]. Specifically, in cancer metabolism, minocycline acts through the inhibition of MMPs [26] or the activation of caspase-induced apoptosis to prevent tumour growth and metastasis [27]. Moreover, minocycline treatment effectively influences the inflammatory process by suppressing the activation of immune cells, inflammation-promoting enzymes, such as iNOS and COX-2, and inflammasomes [28–31]. In addition, minocycline was observed to have modulatory effects by directly targeting the signalling cascade of the mitogen-activated protein kinase (MAPK) pathway or the nuclear factor-κB (NF-κB) pathway [32, 33]. Reports suggest that the pro-inflammatory cytokine TNF-α can activate all the MAPKs, including extracellular signal-regulated kinases (ERK1/2), c-Jun NH2-terminal kinase (JNK) and p38 MAPK [34]. Additionally, NF-κB activation is a main target in the cellular responses to TNF-α [35]. The intracellular signalling pathways by which TNF-α induces transcriptional activity depend on the recruitment of several molecules, for which TNFR-associated factor 2 (TRAF2) was identified as a critical regulator in TNF signal transduction. After TRAF2 receptor recruitment, predominantly to TNF receptor 1 (TNFR1), the signal is conducted through the activation of the IκB kinase (IKK) complex, consisting of IKKα, IKKβ and IKKγ/NEMO, to induce the phosphorylation of IκB, and proteasome-mediated degradation results in the release of the transcription factor NF-κB [36]. However, activation of MAPKs is also mediated by TRAF2, which induces transcriptional activity mainly through the activator protein-1 (AP-1) [36, 37]. Previous reports suggested that the SRC tyrosine kinase acts as a common upstream regulator in the MAPK signalling cascade and that inactivation of SRC also inhibits MAPK signalling processes [38]. Because minocycline interfered with the TNF-α-inducing cell fusion process and caused a dose-dependent decrease in cell fusion events that was most likely attributable to impaired MMP9 expression levels, the minocycline modulating effect along the TNF-mediated signalling pathway was investigated in this study. In general, our data indicated that minocycline is effective in inhibiting TNF-α-induced cell fusion by directly targeting the TNFR1/IKKα/β/IκBα/NFκB signal node to suppress NF-κB inducible target-gene expression of fusion-relevant factors, such as the matrix metalloprotease MMP9.

## Methods

### Cell culture

M13SV1-mCherry-Cre (M13SV1-Cre) human breast epithelial cells and MDA-MB-435-pFDR1 human cancer cells were generated and cultivated as previously described [9, 13]. In brief, M13SV1-mCherry-Cre human breast epithelial cells were derived from M13SV1 human breast epithelial cells [39] by stable transduction with the pcDNA-mCherry-P2A-Cre vector. This vector was derived by excising the mCherry-P2A-Cre sequence of pLM-CMV-R-Cre (a gift from Michel Sadelain (Addgene plasmid # 27546)) with NheI and SalI (both restriction enzymes were purchased from ThermoFisher Scientific GmbH, Schwerte, Germany and cloning it through the pcDNA3.1 vector digested with NheI and XhoI (vector and restrictions enzymes were obtained from ThermoFisher Scientific GmbH, Schwerte, Germany). XhoI and SalI have identical cohesive ends. M13SV1-Cre human breast epithelial cells (M13SV1-Cre cells) were cultivated in MSU-1 basal media (Biochrom GmbH, Berlin, Germany) supplemented with 10% foetal calf serum (FCS; Biochrom GmbH, Berlin, Germany), 1% penicillin and streptomycin (100 U/ml penicillin and 0.1 mg/ml streptomycin; Sigma-Aldrich, Taufkirchen, Germany), 10 μg/ml human recombinant EGF, 5 μg/ml human recombinant insulin, 0.5 μg/ml hydrocortisone, 4 μg/ml human transferrin, 10 nM β-oestrogen (all chemicals were purchased from Sigma-Aldrich, Taufkirchen, Germany) and 1 μg/ml puromycin (InvivoGen, Toulouse, France). MDA-MB-435-pFDR1 cells were generated by stable transfection of MDA-MB-435 cancer cells (HTB 129; LGC Standards GmbH, Wesel, Germany) using the pFDR1 vector (kindly provided by Frank Edenhofer (University of Innsbruck, Innsbruck, Austria)) and were cultivated in DMEM (Sigma-Aldrich, Taufkirchen, Germany) supplemented with 10% FCS (Biochrom GmbH, Berlin, Germany), 1% penicillin and streptomycin (100 U/ml penicillin and 0.1 mg/ml streptomycin; Sigma-Aldrich, Taufkirchen, Germany), and 2 μg/ml puromycin (InvivoGen, Toulouse, France). All cells were maintained in a humidified atmosphere at 37 °C and 5% CO_2_.

### Cell fusion assay

Quantification of fusion events between M13SV1-Cre cells and MDA-MB-435-pFDR1 cells was performed as previously described [9, 13]. The experiment was carried out as a co-culture model at a 1:3 M13SV1-Cre cell to MDA-MB435-pFDR1 cell ratio in a 96-well plate in a humidified atmosphere at 37 °C and 5% CO_2_. As previously described [9, 13], 100 ng/ml TNF-α (Bio-Techne GmbH, Wiesbaden-Nordenstadt, Germany) was added to the co-culture, aligning with the addition of 20 μM minocycline (Sigma-Aldrich, Taufkirchen, Germany). Additionally, for signal transduction studies, inhibitors for the MAPK pathway (MAPKi), as well as inhibitors for the IKK-IκBα signalosome (IKKi), were used. To inhibit the IKK/NF-κB pathway, the IκΒα inhibitor Bay11–7082 and the NF-κB inhibitor PDTC were chosen. The MAPK pathway was blocked with specific MAPKi, including p38-MAPK (SB202190), ERK1/2-MAPK (PD 98,059) and JNK-MAPK (SP600125). The transcription factor AP-1 was blocked with the AP-1 inhibitor SR 11302, whereas SRC kinase was inhibited with PP-1. The various inhibitors were provided as follows: Bay11–7082 and PP-1 were from Merck Millipore (Darmstadt, Germany), PD 98,059 and SB202190 were from Sigma-Aldrich (Taufkirchen, Germany), pyrrolidine dithiocarbamate (PDTC) and SP600125 were from Abcam (Cambridge, UK), and SR 11302 was from ApexBio (Houston, USA). At the end of the co-culture experiment, cells were harvested and washed once with PBS. Finally, the cell fusion events were detected by flow cytometry (FACSCalibur; Becton Dickenson, Heidelberg, Germany), whereby EGFP-positive cells represented the new fusion cells as the result of the Cre-mediated recombination. Freshly harvested M13SV1-Cre cells and MDA-MB-435-pFDR1 cells, mixed at a ratio of 1:3, served as the negative control. The relative fold change was calculated in relation to untreated co-cultured cells, which was set to 1. Each condition was assayed in triplicate.

### RT-PCR and qPCR

For PCR, a total reaction volume of 25 μl was used with ready-to-use 5× Master Mix containing Bio&Sell Taq-polymerase, dNTPs and MgCl2 (Bio&Sell GmbH Nuremberg, Germany) and 10 μM primers (ThermoFisher Scientific GmbH, Schwerte, Germany). Cycling conditions comprising an initial denaturation for 5 min at 94 °C and 30 cycles of 30 s at 94 °C, 30 s at the appropriate annealing temperature and 30 s at 72 °C followed by final elongation for 7 min at 72 °C. The PCR products were separated on 1.5% agarose gel, and bands were visualised with GelRed™ stain (VWR International GmbH, Darmstadt, Germany) with the Gel-Doc™ EZ imaging system (Bio-Rad, Munich, Germany). Real-time PCR was performed to quantify the promoter sequences of MMP9 and ICAM1, which are indicative of κB-binding sites. The following primers were used: forward, 5′-ATTCCCCAGCCTTGCCTA-3′ (− 602), and reverse, 5′-CCTCTTTTTCCCTCCCTGAC-3′ (− 502), for MMP9 and forward, 5′-CAGGGGGCTCATCCACTC-3′ (− 189), and reverse, 5′-TCCATTTCACAAAGCGGTAA-3′ (− 79), for ICAM1. SYBR Green SuperMix with ROX (New England Biolabs GmbH, Frankfurt, Germany) and 10 μM primers were used in a total reaction volume of 10 μl according to the manufacturer’s instructions. The StepOne Plus Real-Time PCR System (ThermoFisher Scientific GmbH, Schwerte, Germany) was used for qPCR. NF-κB-p65 enrichment was determined in relation to input DNA. The results are presented as fold changes normalised to IgG and in comparison to the untreated control cells.

### Western blot analysis

M13SV1-Cre cells and MDA-MB-435-pFDR1 cells were seeded at a density of 0.5 to 1 × 10^6^ cells in 25 cm^2^ flasks and cultivated at 37 °C and 5% CO_2_ overnight. At 80% confluency, cells were pre-treated with different concentrations of minocycline (20 μM or 100 μM) for a total of 24 h or 72 h, respectively. Cells were stimulated with TNF-α (100 ng/ml) for 2 h at 37 °C and 5% CO_2_ or simultaneously with 20 μM minocycline for 72 h. Cells were harvested, subjected to sedimentation by centrifugation (10 min and at 340×g) and lysed in ice-cold RIPA buffer (50 mM Tris-HCl pH 8.0; 150 mM NaCl, 1% (v/v) NP-40, 0.5% (w/v) sodium deoxycholate, 0.1% (w/v) and sodium dodecyl sulphate) supplemented with Complete, Mini, EDTA-free Protease Inhibitor Cocktail (Sigma-Aldrich, Taufkirchen, Germany) and Pierce Phosphatase Inhibitor Mini Tablets (ThermoFisher Scientific GmbH, Schwerte, Germany). The samples were sonicated three times (10 s on and 30 s off), and the total protein concentration was determined using the Pierce™ BCA Protein Assay Kit (ThermoFisher Scientific GmbH, Schwerte, Germany) according to the manufacturer’s instructions. For western blotting, samples complemented with 3× Laemmli sample buffer were denatured by boiling at 95 °C for 6 min. A total protein fraction of 40 μg was subjected to SDS-PAGE, with 10–15% polyacrylamide gel and transferred to an Immobilon polyvinyl difluoride (PVDF) nitrocellulose membrane (Merck Millipore, Darmstadt, Germany) under semi-dry conditions. Membranes were incubated in 5% (w/v) BSA or 5% (w/v) non-fat milk powder (Applichem, Darmstadt, Germany) in PBS-T (phosphate-buffered saline) with 0.1% (v/v) Tween 20 (PBS-T) for 1 h at room temperature. For antibody incubation, blocking buffer specific for each antibody was added to the PVDF membranes, which were rolled in the antibody-blocking solution overnight at 4 °C. The antibodies used for the western blot analysis are listed in Table 1.

**Table 1:** Antibodies used in this study

Antibody	Source	Clone	Company
anti-NF-κB p65	rabbit monoclonal	D14E12	Cell Signaling^a^
anti-phospho-NF-κB p65(S536)	rabbit monoclonal	93H1	Cell Signaling^a^
anti-SRC	rabbit monoclonal	36D10	Cell Signaling^a^
anti-phospho-SRC (Tyr416)	rabbit polyclonal		Cell Signaling^a^
anti-IKK-α	rabbit polyclonal		Cell Signaling^a^
anti-IKK-β	rabbit monoclonal	2C8	Cell Signaling^a^
anti-phospho-IKK-α/β (S176/180)	rabbit monoclonal	16A6	Cell Signaling^a^
anti-IκBα	rabbit monoclonal	44D4	Cell Signaling^a^
anti-phospho-IκBα (S32)	rabbit monoclonal	14D4	Cell Signaling^a^
anti-SAPK/JNK	rabbit polyclonal		Cell Signaling^a^
anti-phospho-SAPK/JNK(T183/Y185)	mouse monoclonal	G9	Cell Signaling^a^
anti-p38 MAPK	rabbit polyclonal		Cell Signaling^a^
anti-phospho-p38 MAPK (T180/Y182)	rabbit monoclonal	3D7	Cell Signaling^a^
anti-p44/42 MAPK (Erk1/2)	rabbit polyclonal		Cell Signaling^a^
anti-phospho-p44/42 MAPK (T202/Y204)	rabbit polyclonal		Cell Signaling^a^
anti-TNFR1	rabbit monoclonal	C25C1	Cell Signaling^a^
anti-TRAF2	rabbit monoclonal	C192	Cell Signaling^a^
anti-eIF4E	rabbit monoclonal	46H6	Cell Signaling^a^
anti-β-actin	mouse monoclonal	AC-74	Sigma-Aldrich^b^
ALEXA488-conjugated goat-anti-rabbit IgG (H + L)	rabbit polyclonal		ThermoFisher Scientific GmbH^d^
anti-mouse-IgG-HRP	mouse polyclonal		Cell Signaling^a^
anti-rabbit-IgG-HRP	rabbit polyclonal		Cell Signaling^a^
anti-rabbit IgG light-chain specific-HRP	mouse monoclonal	D4W3E	Cell Signaling^a^
IgG	rabbit polyclonal		Merck^c^

^a^ Cell Signaling Technology Europe B.V., Frankfurt am Main, Germany; ^b^ Sigma-Aldrich, Taufkirchen, Germany; ^c^ Merck, Darmstadt, Germany; ^d^ ThermoFisher Scientific GmbH, Schwerte, Germany

### ChIP assay

A ChIP assay was performed to examine the NF-κB-binding activity of the selected promotor regions of MMP9 and ICAM1. Therefore, M13SV1-Cre cells and MDA-MB435-pFDR1 cells were pre-treated with minocycline (100 μM) for 24 h. Subsequently, TNF-α (100 ng/ml) was added to the cells for an additional 2 h. Control samples with and without minocycline (100 μM) or TNF-α (100 ng/ml) were treated identically to the minocycline + TNF-α treatment. The following steps were performed according to the manufacturer’s instructions (ChIP-IT Express®, Active Motif, La Hulpe, Belgium). First, cells with a density of approximately 80% were fixed with 1% paraformaldehyde (Affymetrix, Ohio, USA) for 10 min, and then, to stop the reaction immediately, glycine stop-fix solution was used. Then, the cells were carefully scraped into a cell-scraping solution and collected in 1.5 ml Eppendorf tubes. After centrifugation (for 10 min at 720×g and 4 °C), cells were lysed in lysis buffer with PIC and PMSF for 30 min on ice. Subsequently, the chromatin was sonicated to an average size of 0.2–0.5 kb in a Bioruptor UCD-300 TM (Diagenode, Liège, Belgium). For immunoprecipitation, equal amounts of the sheared chromatin were incubated with an anti-NF-κB-p65 antibody (Table 1) or the IgG control antibody (Table 1) together with 30 μl of protein G magnetic beads (Active motif, La Hulpe, Belgium) for 8 h at 4 °C with mild rotation. One of the sheared chromatin aliquots was saved in a freezer at − 20 °C until it was precipitated for use as the input control. The immunocomplexes were selected by magnetic separation, which included three washing steps. The chromatin was acquired by elution from the beads and the crosslinks were reversed by incubation of samples in reverse cross-linking buffer for 15 min at 95 °C in a thermocycler while shaking, followed by proteinase digestion with proteinase K for an additional 1 h at 37 °C. The enriched DNA was extracted by 1:1 phenol:chloroform (Sigma-Aldrich, Taufkirchen, Germany) and precipitated by acetate (pH 5.2) and 100% ethanol. For downstream assays, PCR and qPCR were performed, to amplify the promotor regions of MMP9 (− 602/− 502) and ICAM1 (− 189/− 79), which are known to contain the κB sites [40, 41].

### Co-immunoprecipitation

M13SV1-Cre cells and MDA-MB435-pFDR1 cells were pre-treated with minocycline (100 μM) for 24 h at 37 °C and 5% CO_2_ in a humidified atmosphere. Then, both cell types were stimulated with TNF-α (100 ng/ml) for 1 h_._ Cells were washed once in PBS and scraped in ice-cold lysis buffer (1% (v/v) NP-40, 50 mM Tris-HCl (pH 7.5), 150 mM NaCl, 1 mM EDTA, and 1 mM EGTA) and a proteinase inhibitor cocktail (see western blot protocol) for 30 min on ice. After being subjected to sonification three times (5 s on and 20 s off) at the lowest pulse frequency, samples were centrifuged (10 min, 340×g) to remove cell debris. The debris-free supernatants were collected in new 1.5 ml collection tubes. The total protein concentration of each sample was determined by using the Pierce™ BCA Protein Assay Kit (ThermoFisher Scientific GmbH, Schwerte, Germany) according to the manufacturer’s instructions. A final concentration of 500 μg protein was used for the Co-IP experiment. First, cell lysates containing 500 μg of protein were cleaned by pre-treatment with 25 μl of Protein A Magnetic Beads (Cell Signaling, Leiden, Netherlands) for 2 h at 4 °C with mild rotation. After magnetic separation, lysates were incubated with anti-TNFR1 antibody (Table 1) or IgG antibody (Table 1) at 4 °C for 18–24 h. Then, 30 μl of magnetic beads was mixed with the lysates for an additional 2 h at 4 °C. Immunocomplexes were separated on a magnetic rack and washed three times in lysis buffer to loaded them with 3× Laemmli sample buffer (with DTT but without β-mercaptoethanol) and boiled at 95 °C for 5 min. Samples were loaded on 12% gel for SDS-PAGE, and western blotting was completed as described. The antibody incubation was carried out in 5% (w/v) non-fat milk-blocking solution overnight at 4 °C. Then, secondary antibody was added to the membrane for an additional 1 h, using the anti-rabbit IgG light-chain mouse (HRP) antibody (Table 1) to prevent the detection of the unspecific bound heavy-chain antibody at the 55 kDa level. The antibodies used for western blot analysis are listed in Table 1.

### Immunocytochemical staining

The cytosolic and nuclear locations of the NF-κB-p65 transcription factor were detected by using a Leica TCS SP5 confocal laser scanning microscope (Leica, Wetzlar, Germany). Cells were seeded at a concentration of 2 × 10^4^ in a chamber slide (ThermoFisher Scientific GmbH, Schwerte, Germany) at 37 °C and 5% CO_2_. Minocycline (100 μM) or PDTC (50 μM) was added to the cells, and incubation was carried out for an additional 24 h. After TNF-α (100 ng/ml) stimulation for 2 h at 37 °C and 5% CO_2_, the cells were fixed with 4% paraformaldehyde solution (Affymetrix, Ohio, USA) for 10 min at RT and washed twice with PBS. Permeabilisation was performed for an additional 5 min with 0.5% Triton X (AppliChem, Darmstadt, Germany), followed by three washes. Then, cells were blocked in 1.5% BSA/PBS for 30 min at RT. Cells were incubated with a specific NF-κB p65 antibody (Table 1) or with the corresponding IgG control antibody (Table 1), diluted 1:400 in 1.5% BSA/PBS for 1 h at RT and washed again with PBS. The secondary antibody, ALEXA488-conjugated goat-anti-rabbit IgG (H + L) (Table 1), was added at a concentration of 1:500 in PBS for an additional 45 min at RT and without light. The antibody solution was removed, and the cells were rewashed another three times in PBS. The nuclei were stained with propidium iodide (PI) 1:2000 (ThermoFisher Scientific GmbH, Schwerte, Germany) for 1 min. Excess PI was removed by thorough washing with PBS (two times) and H_2_O (once). Cells were mounted in Fluoromount™ aqueous mounting media (Sigma-Aldrich, Taufkirchen, Germany).

### Preparation of cytosolic and nuclear extracts

The cell extracts containing nuclei and cytosol were recovered by using the NE-PER Nuclear and Cytosolic Extraction Kit (ThermoFisher Scientific GmbH, Schwerte, Germany) according to the manufacturer’s instructions. In brief, cells were prepared as previously described with or without minocycline (100 μmol/l) and with or without TNF-α (100 ng/ml). After the specific treatment, cells were harvested and lysed in ice-cold Cytoplasmic Extraction Reagent I (CER I) buffer with proteinase inhibitors (Sigma-Aldrich, Taufkirchen, Germany) for 10 min on ice. Subsequently, CER II buffer was added to the lysates, and after subjected to a vigorous mixing for 5 s and incubation on ice for 1 min, the cell lysates were pelleted by centrifugation (5 min at 13,000×g). Thereafter, supernatants were stored at − 80 °C (cytosolic extract), and the pellets were resuspended in ice-cold nuclear extraction buffer (NER) with proteinase inhibitors. The nuclear fraction was prepared by vortexing the samples at the highest setting for 15 s every 10 min, and it was incubated on ice during the breaks from mixing. After another centrifugation step (13,000×g for 15 min), nuclear extracts were collected and stored at − 80 °C until use. Protein concentrations were determined using the Pierce™ BCA Protein Assay Kit (ThermoFisher Scientific GmbH, Schwerte, Germany), and western blotting was performed as described above.

### Luciferase reporter assay

To measure the influence of minocycline on NF-κB activation, the luciferase reporter vector pHAGE-NFkB-TA-LUC-UBC-GFP-W (a gift from Darrell Kotton (Addgene plasmid # 49343; http://n2t.net/addgene:49343) [42]), which is responsive to NF-κB, was transiently transfected into M13SV1-Cre cells. The accuracy of the Luciferase reporter construct was confirmed by using the control Renilla luciferase reporter pcDNA-REN. This vector was originally derived from the pIS1 plasmid (a gift from David Bartel (Addgene plasmid # 12179; http://n2t.net/addgene:12179)). The Renilla luciferase gene was excised by KpnI and NotI digestion (both restriction enzymes were purchased from ThermoFisher Scientific GmbH, Schwerte, Germany) and cloned into the pcDNA3.1 vector (ThermoFisher Scientific GmbH, Schwerte, Germany). The cells were cultivated in 6-well plates for 24 h to reach a confluency of nearly 80%. Then, cells were co-transfected with 4 μg pHAGE-NFkB-TA-LUC-UBC-GFP-W plasmid and 1 μg pcDNA-REN control plasmid by lipofection (Lipofectamine™ 2000; ThermoFisher Scientific, Schwerte, Germany) as described in the user’s manual. The next day, the successful transfection levels, as EGFP-positive cells, were determined by fluorescence microscopy, and 1 × 10^4^ cells/well were seeded in 96-well plates for an additional 24 h in a humidified atmosphere. After 24 h post-transfection, cells were incubated with different concentrations of minocycline (50 μM or 100 μM) or with 50 μM PDTC and cultivated for an additional 24 h. The cells were then stimulated with TNF-α (100 ng/ml) for 2 h, and luciferase activity was measured by using the Dual-Glo® Luciferase Assay System (Promega, Mannheim, Germany) according to the manufacturer’s instructions. Non-transfected served as a control and were set to 1. Each condition was assayed in triplicate, and detection was performed by using a Mithras LB 940 luminometer (Berthold Technologies; Bad Wildbad, Germany).

### Statistical analysis

Statistical significance of the data was calculated by the ANOVA F-test. Multiple comparisons were conducted using Scheffé post hoc tests. The Kruskal-Wallis test was used for the evaluation of the ChIP data. Statistical analyses were performed using SPSS Version 24.0.0.1, and *p*-values < 0.05 were considered indicators of significance.

### Language style editing

This manuscript was language edited by American Journal Experts, Durham, NC, USA.

## Results

### Minocycline inhibits TNF-α-induced TNFR1-TRAF2 receptor association

**Fig. 1:**
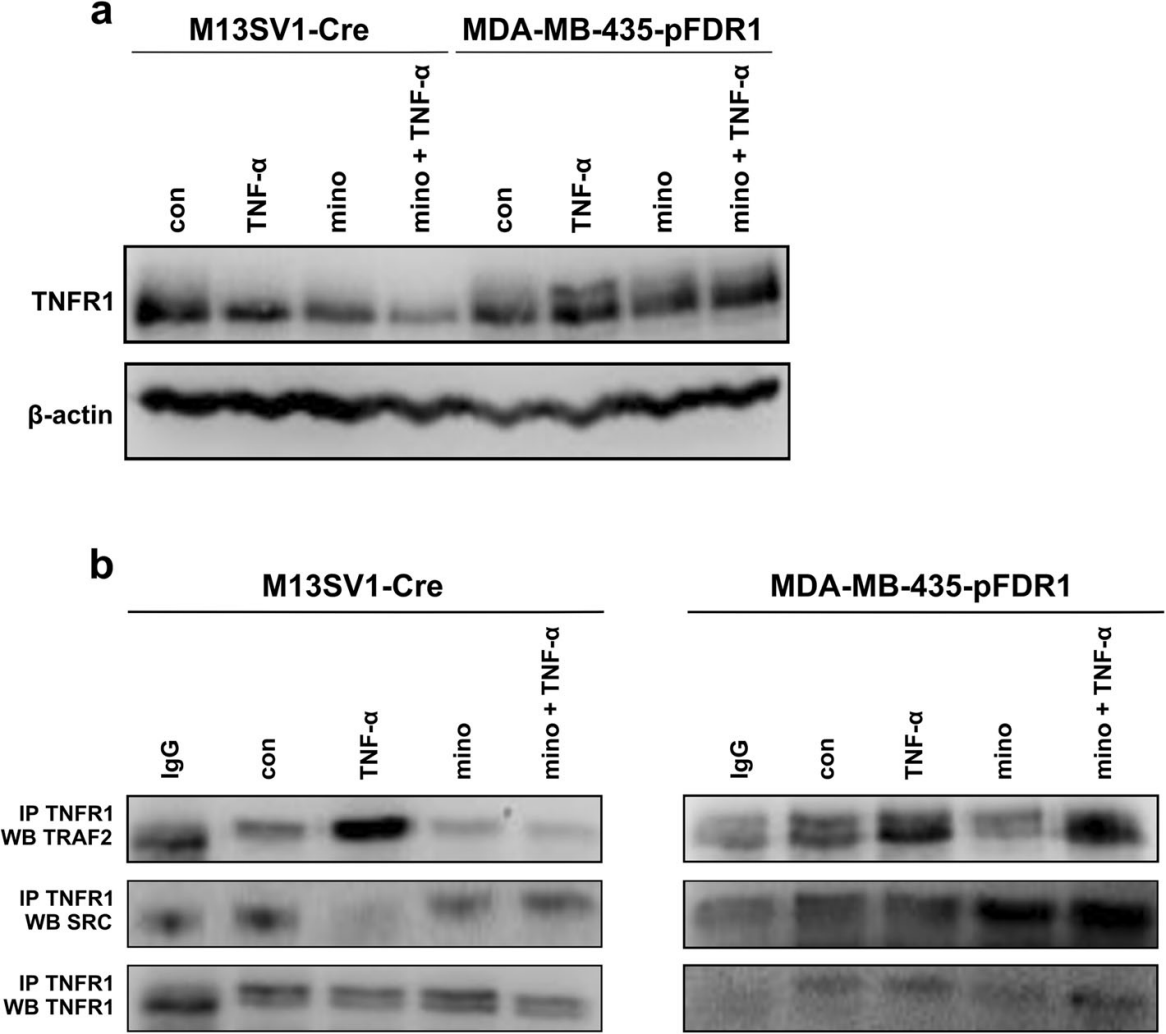
Minocycline inhibits TNF-α-induced TRAF2-TNFR1 receptor binding. M13SV1-Cre cells and MDA-MB-435-pFDR1 cells were pre-treated with 100 μM minocycline for 24 h. Cells were additionally stimulated with 100 ng/ml TNF-α either for 2 h for western blot analysis (**a**) or for 1 h for co-immunoprecipitation studies (**b**). Co-immunoprecipitation was either performed using a TNFR1 antibody or a normal anti-rabbit IgG antibody. Western blotting was carried out by using the TRAF2 or the SRC antibody. Representative data from at least three independent experiments are shown

Since we have recently shown that the TNF-α-induced cell fusion process between M13SV1-Cre breast epithelial cells and human MDA-MB-435-pFDR1 breast cancer cells is abolished by minocycline [13], we wondered how minocycline might affect TNF-α-induced signal transduction pathways in detail and which of them are relevant in promoting the cell fusion step. It is well known that two TNF-α receptors exist: TNFR1 and TNFR2 [43]. However, the blockade of TNFR1 by using specific antibodies against TNFR1 was more effective than TNFR2 blocking in decreasing cell fusion events [9], and therefore, we decided to investigate only the TNFR1-dependent signal transduction pathway in both cell lines. Addition of TNF-α, minocycline and a combination of both to the cells revealed that TNFR1 expression was decreased in the presence of minocycline in M13SV1-Cre cells but remained unchanged in MDA-MB-435-pFDR1 cells (Fig. 1a). Because TNFR1 is primarily involved in the TNF-α-induced signal transduction pathway, the recruitment of the adaptor proteins TRAF2 and SRC kinase to TNFR1 after TNF-α stimulation in minocycline-saturated cells was investigated. As shown in Fig. 1b, an increased TNF-α induced TRAF2 recruitment to TNFR1 was observed in both cell lines, which was impaired by minocycline in M13SV1-Cre cells but not in MDA-MB-435-pFDR1 cells.

Moreover, TNFR1-SRC binding was examined as it was suspected to mediate the activation of MAP kinases JNK, ERK1/2 and p38 [44]. In contrast to TRAF2, the TNFR1-SRC association could not be detected in M13SV1-Cre cells after TNF-α stimulation, while in minocycline-treated cells, the effect was not observed. Interestingly, the TNF-α blocking effect of SRC on TNFR1 was completely abrogated in M13SV1-Cre cells pre-incubated with minocycline. In contrast to that in M13SV1-Cre cells, the TNFR1-SRC association was improved by minocycline and minocycline with TNF-α in MDA-MB-435-pFDR1 cells.

### Minocycline impairs TNF-α-induced activation of MAP kinases p38, JNK and ERK1/2

**Fig. 2:**
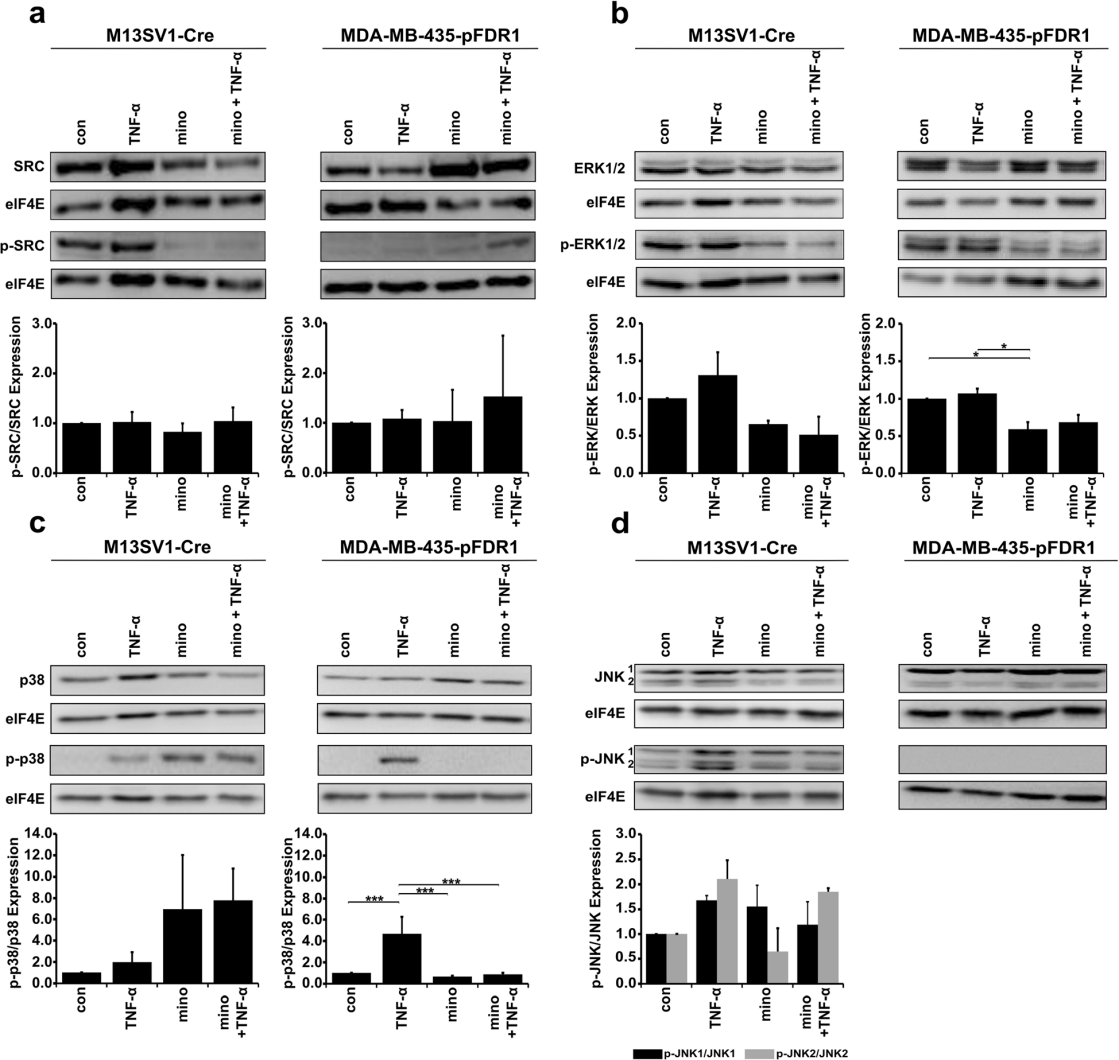
Minocycline impairs the TNF-α-induced activation of SRC kinase and of MAPKs. M13SV1-Cre cells and MDA-MB-435-pFDR1 cells were treated with or without TNF-α (100 ng/ml) and with or without minocycline (20 μM) for 72 h. Whole cell lysates were collected, and constitutive and phosphorylated protein levels were analysed by western blotting: **a** SRC/ phos-SRC, **b** ERK1/2/ phos-ERK1/2, **c** p38/ phos-p38, and **d** JNK/ phos-JNK. Relative native and phosphorylated protein levels were normalised to the level of housekeeping gene expression and in relation to the control, which was set to 1. Representative data from at least three independent experiments are shown. Statistical analysis: ANOVA F-test and Scheffé post hoc test: * = *p* < 0.05; ** = *p* < 0.01; *** = *p* < 0.001

First, the activation of the SRC kinase was examined to determine whether SRC kinase activation was also linked to MAPK activation. TNF-α induction did not activate SRC kinase in either cell line (Fig. 2a). Western blot data revealed that minocycline treatment reduced total SRC expression in M13SV1-Cre cells, which subsequently led to a lower activation level of SRC (Fig. 2a). This result contradicts the finding for MDA-MB-435-pFDR1 cells, in which total SRC expression and activation levels were enhanced upon minocycline treatment (Fig. 2a). Second, the activation of the MAPK members ERK1/2, JNK and p38 was investigated. The data showed that all MAPK members were activated in TNF-α-stimulated M13SV1-Cre cells (Fig. 2b-d). Interestingly, although minocycline effectively impaired SRC kinase, ERK1/2 and JNK phosphorylation in M13SV1-Cre cells markedly increased phosphorylated p38 levels observed (Fig. 2a-d). In MDA-MB-435-pFDR1 cells, minocycline suppressed TNF-α-induced phosphorylation of both ERK1/2 and p38, while JNK phosphorylation was not detected (Fig. 2b-d).

### TNF-α-induced cell fusion between M13SV1-Cre and MDA-MB-435-pFDR1 cells is independent of JNK, ERK1/2, p38, and SRC kinase signalling and AP-1 function

**Fig. 3:**
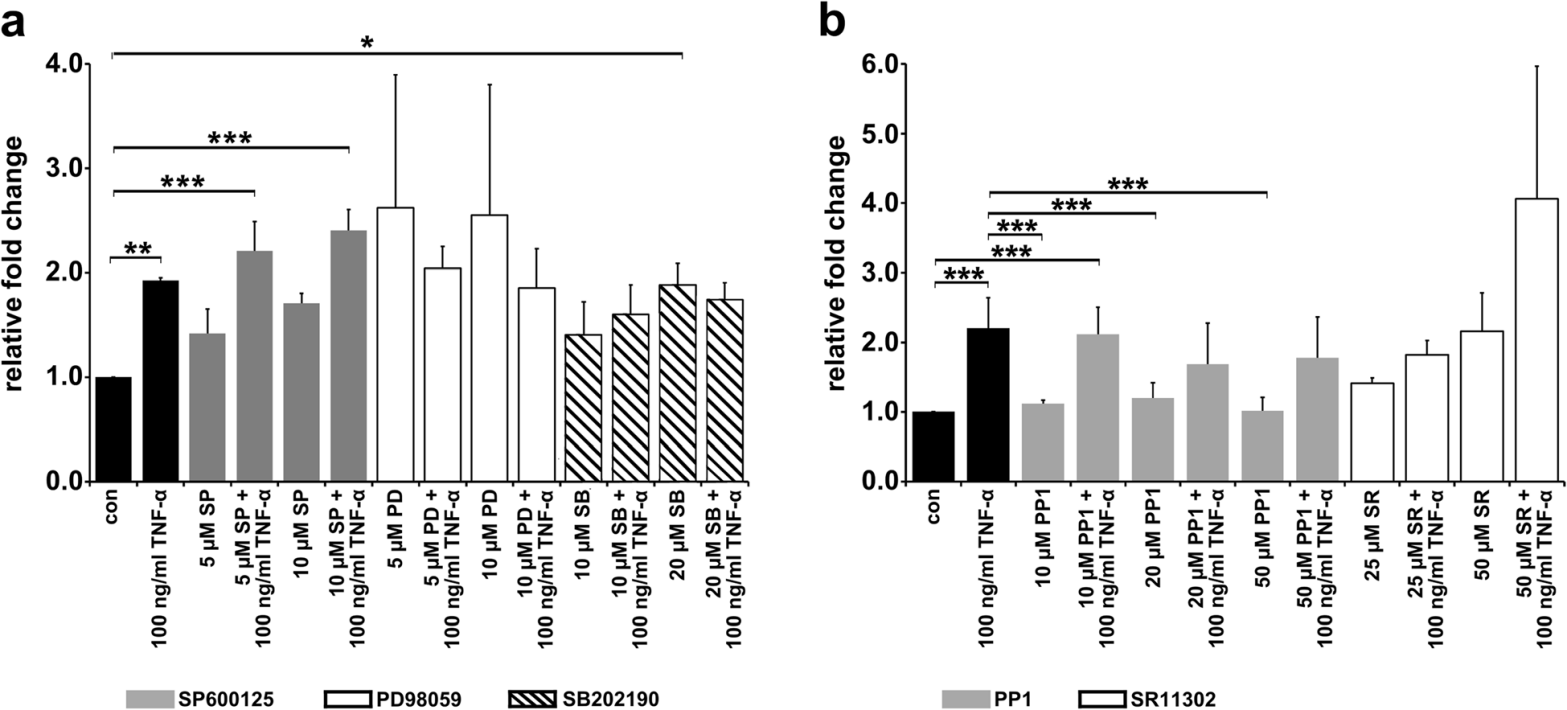
TNF-α induced cell fusion is not affected by ERK1/2, p38, JNK, SRC or AP-1 inhibition. Cell fusion was carried out in the presence of 100 ng/ml TNF-α and different concentrations of selective inhibitors. **a** Data for SP600125 (SP; JNK inhibitor), PD98059 (PD; Erk1/2 inhibitor) and SB202190 (SB; p38 inhibitor). **b** Results for PP1 (SRC inhibitor) and SR (SR11302; AP-1 inhibitor). The results are presented as the means ± STD of at least three independent experiments. Statistical analysis: ANOVA F-test and Scheffé post hoc test: * = *p* < 0.05; ** = *p* < 0.01; *** = *p* < 0.001

Next, cell fusion frequency in the presence of different MAPK inhibitors was measured to clarify which of the MAPK signalling pathways might be involved in the TNF-α-induced cell fusion process. Cells were treated with different concentrations of inhibitors: SP600125 for JNK inhibition, PD98059 for ERK1/2 inhibition and SB202190 for p38 inhibition in addition to TNF-α for 72 h (Fig. 3a). Interestingly, inhibition of MAPK signalling was rather associated with an increased cell fusion frequency of the cells. Compared to untreated and TNF-α stimulated cells dose-dependent higher cell fusion numbers were observed for SP600125 and SP600125 + TNF-α treated cells. The addition of the ERK1/2 inhibitor PD98059 led to a large increase in cell fusion events, which seems to be blocked in the presence of TNF-α. Inhibition of p38 resulted in a dose-dependent increase in cell fusion, similar to that by TNF-α induction, but without any additional effect. Furthermore, SRC kinase activation was blocked using the SRC inhibitor PP1. A slightly decreased TNF-α-induced cell fusion frequency was observed in the presence of 20 μM and 50 μM PP1 (Fig. 3b). Because the transcription factor AP-1 is one of the main targets in the downstream MAPK signalling cascade, we also tested whether AP-1 inhibition was influenced by the TNF-α-induced cell fusion process. In this case, we found that, up to a concentration of 25 μM, no effect on the TNF-α-induced fusion process could be observed. In contrast, markedly increased cell fusion rates were observed with the addition of 50 μM SR11302 and 50 μM S211302 + 100 ng/ml TNF-α (Fig. 3b). These results implicate MAPK signalling activity in the negative regulation of cell fusion, which was indicated by all the MAPKs being blocked, and therefore, AP-1 activity ceasing, thus leading to a higher cell fusion frequency.

### Minocycline impairs TNF-α-induced activation of IKK-IκBα signalling

Because TNF-α signalling leads to the activation of the IKK complex, we analysed the phosphorylation state of the IKKα/β/IκBα protein complex. In brief, the data showed that minocycline had no inhibitory effect as it failed to block TNF-α-induced phosphorylation of the IKKα/β complex. It was observed that total IKKα expression was reduced by minocycline in both cell lines, while constitutive IKKβ expression seems to be unaltered by minocycline. In addition, minocycline suppressed TNF-α-induced IκBα phosphorylation in M13SV1-Cre cells but not in MDA-MB-435-pFDR1 cells. Specifically, we observed slightly increased phosphorylated IκBα levels in MDA-MB-435-pFDR1 cells co-treated with minocycline and TNF-α. These data suggest that minocycline modulated the activation of IκBα differently in the two cell lines. Hence, minocycline might act at the IκBα level, which is necessary for its degradation and for the release of transcription factor NF-κB.

### TNFα-induced cell fusion depends on activation of the NF-κB pathway

**Fig. 4:**
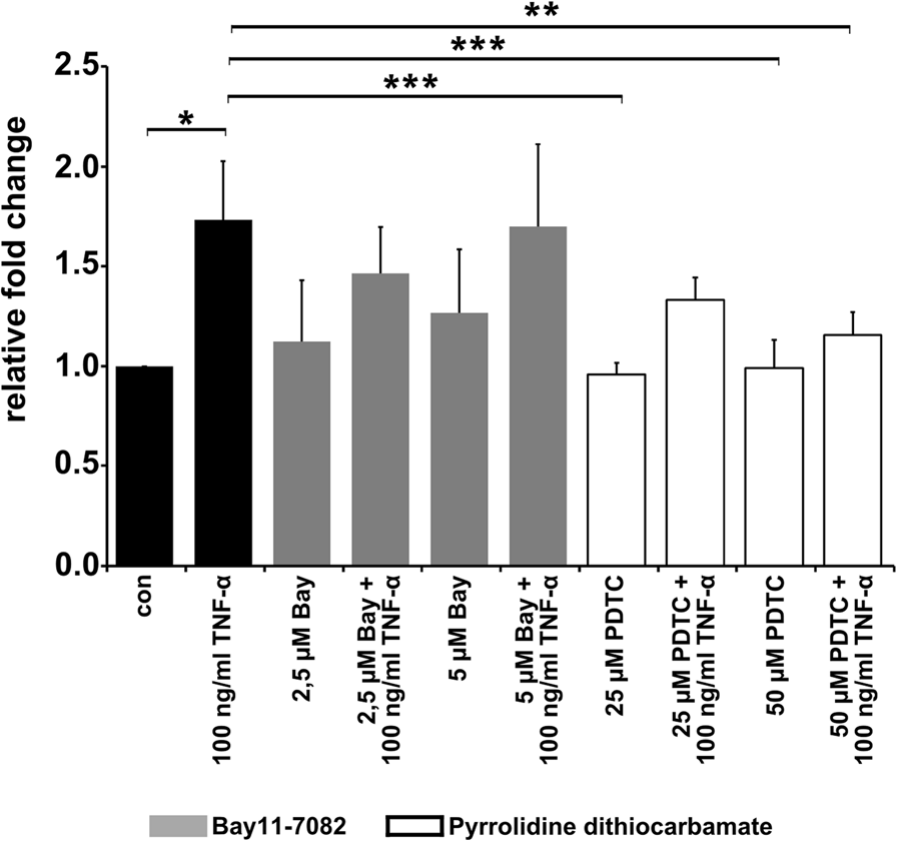
TNF-α-induced cell fusion depends on the activation of the NF-κB pathway. Cells were treated with TNF-α (100 ng/ml) and either IκBα inhibitor Bay11–7082 (Bay) or NF-κB inhibitor PDTC for 72 h. The results are shown as the means ± STD of at least three independent experiments. Statistical analysis: ANOVA F-test and Scheffé post hoc test: * = *p* < 0.05; ** = *p* < 0.01; *** = *p* < 0.001

**Fig. 5:**
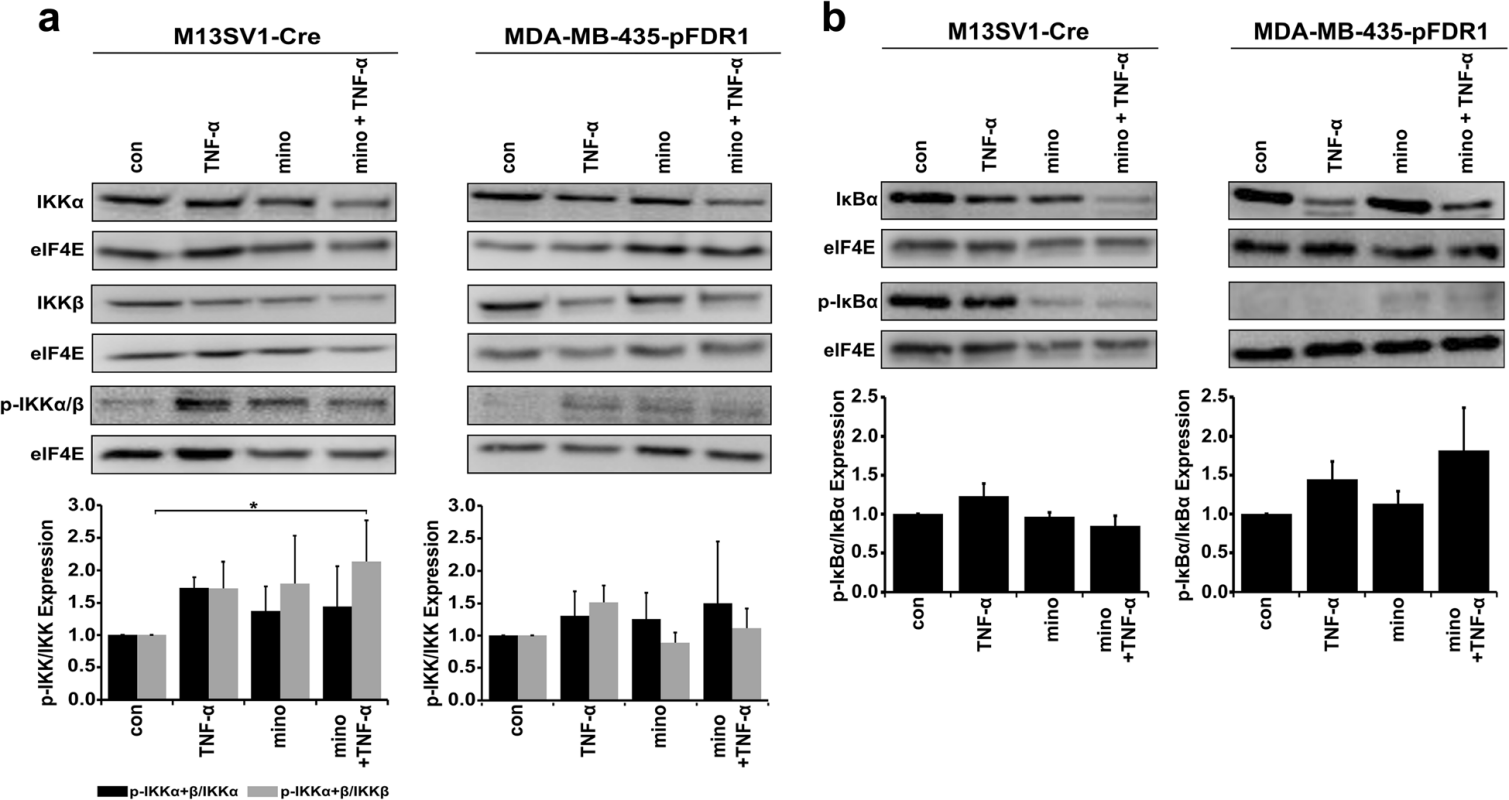
Minocycline impairs the TNF-α-induced activation of IKK-IκBα signalling. Cells were stimulated with or without TNF-α (100 ng/ml) and minocycline (20 μM) for 72 h to analyse the phosphorylation of (**a**) IKKα/β and (**b**) IκBα by western blotting. Relative native and phosphorylated protein levels were normalised to the level of housekeeping gene expression and in relation to control, which was set to 1. Representative data from at least three independent experiments are shown. Statistical analysis: ANOVA F-test and Scheffé post hoc test: * = *p* < 0.05; ** = *p* < 0.01; *** = *p* < 0.001

We investigated whether activation of IκB kinase signalling is as important as it is suspected of being. To this end, we used specific inhibitors targeting the IκBα protein (Bay11–7082) or NF-κB (PDTC) and performed cell fusion experiments. Interestingly, the inhibition of only NF-κB was correlated with a decreased TNF-α-induced cell fusion rate, whereas no inhibitory effect was observed for IκBα inhibition by Bay 11–7082 (Fig. 4). Although the IC50 of Bay11–7082 is approximately 10 μM, we decided to use a concentration of 5 μM because of the cytotoxic effects that emerged during an incubation time of 72 h (data not shown). However, the Bay-7082 fusion data were opposite to those shown in Fig. 5b; that is, IκBα expression levels and phosphorylated IκBα levels were diminished in M13SV1-Cre cells treated with TNF-α and minocycline. These results confirmed that the pro-fusogenic effects of TNF-α depend on the activation of transcription factor NF-κB.

### Minocycline inhibits TNF-α-induced phosphorylation and nuclear translocation of NF-κB-p65 in M13SV1-Cre cells

**Fig. 6:**
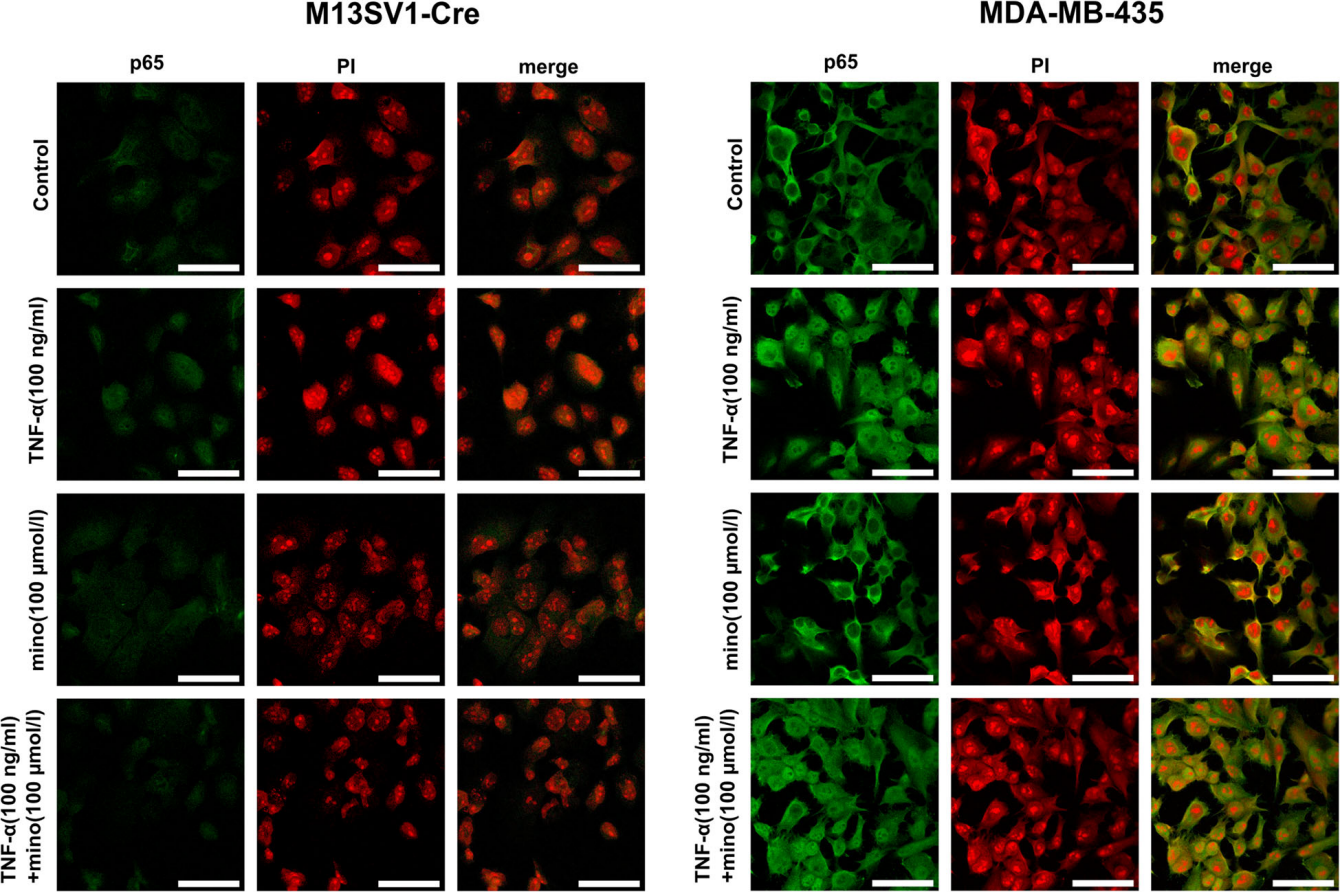
Minocycline inhibits TNF-α-induced NF-κB nuclear translocation in M13SV1-Cre cells but not in MDA-MB-435 cells. Cells were pre-treated with 100 μM minocycline for 24 h and then with 100 ng/ml TNF-α for an additional 2 h. Instead of MDA-MB-435-pFDR1 cells, wild-type MDA-MB-435 cells were used, which do not express the DsRed fluorescent protein that interferes with p65 and PI staining. Representative images of at least three independent experiments are shown. Bar = 50 μm

**Fig. 7:**
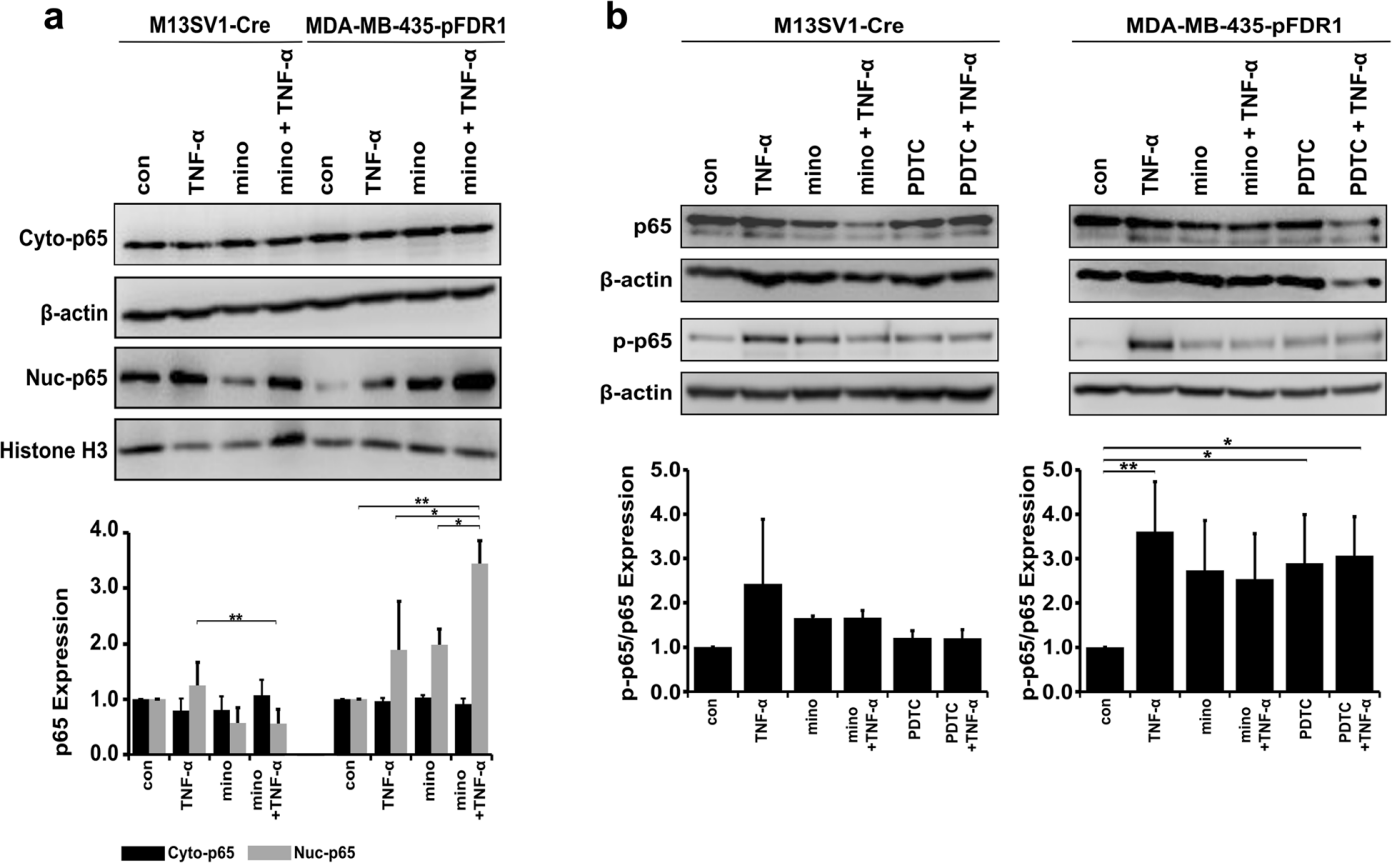
Minocycline inhibits NF-kB-p65 nuclear translocation/ activation in M13SV1-Cre cells but not in MDA-MB-435-pFDR1 cells. **a** Cytoplasmic and nuclear cell lysate extracts of M13SV1-Cre and MDA-MB-435-pFDR1 cells under control conditions, TNF-α (100 ng/ml) induction, minocycline treatment (100 μM) or minocycline + TNF-α exposure were collected and analysed by western blotting. **b** PDTC (50 μM) was used as an additional control to block NK-κB. Relative NF-κB p65 protein and phosphorylated protein levels in the cytoplasm and in the nucleus were normalised to the levels of β-actin and histone H3, respectively, and in relation to control, which was set to 1. Representative western blot data of at least three independent experiments are shown. Statistical analysis: ANOVA F-test and Scheffé post hoc test: * = *p* < 0.05; ** = *p* < 0.01; *** = *p* < 0.001

Based on previously generated data, the effect of minocycline on the TNF-α mediated signalling process was further examined through observations of the transcription factor NF-κB directly. For these observations, cells were cultured with 100 μM minocycline for 24 h and then stimulated by TNF-α (100 ng/ml) for additional 2 h. The nuclear translocation of the NF-κB subunit p65 was detected using immunochemistry (Fig. 6). To quantify nuclear accumulation of NF-κB-p65, cytosolic and nuclear protein fractions of the cells were analysed by western blotting (Fig. 7a). Comparable cytosolic NF-κB-p65 levels were determined in both untreated and treated cells of both cell lines (Fig. 7a). Immunofluorescence staining of the p65 subunit indicated that TNF-α increased nuclear NF-κB-p65 levels in both cell lines, and this increase could be suppressed by the administration of minocycline in M13SV1-Cre cells but not in MDA-MB-435-pFDR1 cells (Fig. 6). Western blot results were in line with the staining data. That is, slightly increased nuclear NF-κB levels were observed in TNF-α-stimulated M13SV1-Cre cells but were not observed in minocycline and minocycline + TNF-α-treated cells (Fig. 7a). In contrast to M13SV1-Cre cells, TNF-α, minocycline and minocycline + TNF-α stimulation of MDA-MB-435-pFDR1 cells resulted in an increased amount of NF-κB p65 in the nucleus (Fig. 7a), a finding in agreement with increased phosphorylated IκBα levels that were observed in minocycline and minocycline + TNF-α treated MDA-MB-435-pFDR1 cells (Fig. 5b). Furthermore, the activation level of NF-κB-p65 was examined to determine whether minocycline administration also influenced the phosphorylation of the NF-κB p65 subunit (Fig. 7b). Because NF-κB-p65 transactivation is associated with IKK-dependent phosphorylation at the serine residue 536 [45], we used a specific antibody that enabled us to determine the phosphorylation at Ser-536 of p65. Additionally, PDTC was used as a negative control because of its pharmacological potency in inhibiting NF-κB-p65 activation. It was observed that phosphorylation of NF-κB-p65 was increased after TNF-α stimulation in both cell lines, and it was inhibited by minocycline in M13SV1-Cre cells. In MDA-MB-435-pFDR1 cells, minocycline, minocycline + TNF-α and minocycline + PDTC induced an increase in the phosphorylation of Ser-536 of NF-κB-p65 compared to the control group (Fig. 7b). However, the most intensive signal was detected in TNF-α-treated MDA-MB-435-pFDR1 cells. These findings indicate that minocycline was sufficient to inhibit the TNF-α-induced activation of NF-κB by suppressing phosphorylation of Ser-536 and nuclear translocation in M13SV1-Cre cells. This finding is in contrast to that for MDA-MB-435-pFDR1 cells, in which minocycline seems to act as an inducer of NF-κB-p65 Ser-536 phosphorylation as well as in its nuclear translocation.

### Minocycline impairs NF-κB-dependent transcription of MMP9 and ICAM1 target genes

**Fig. 8:**
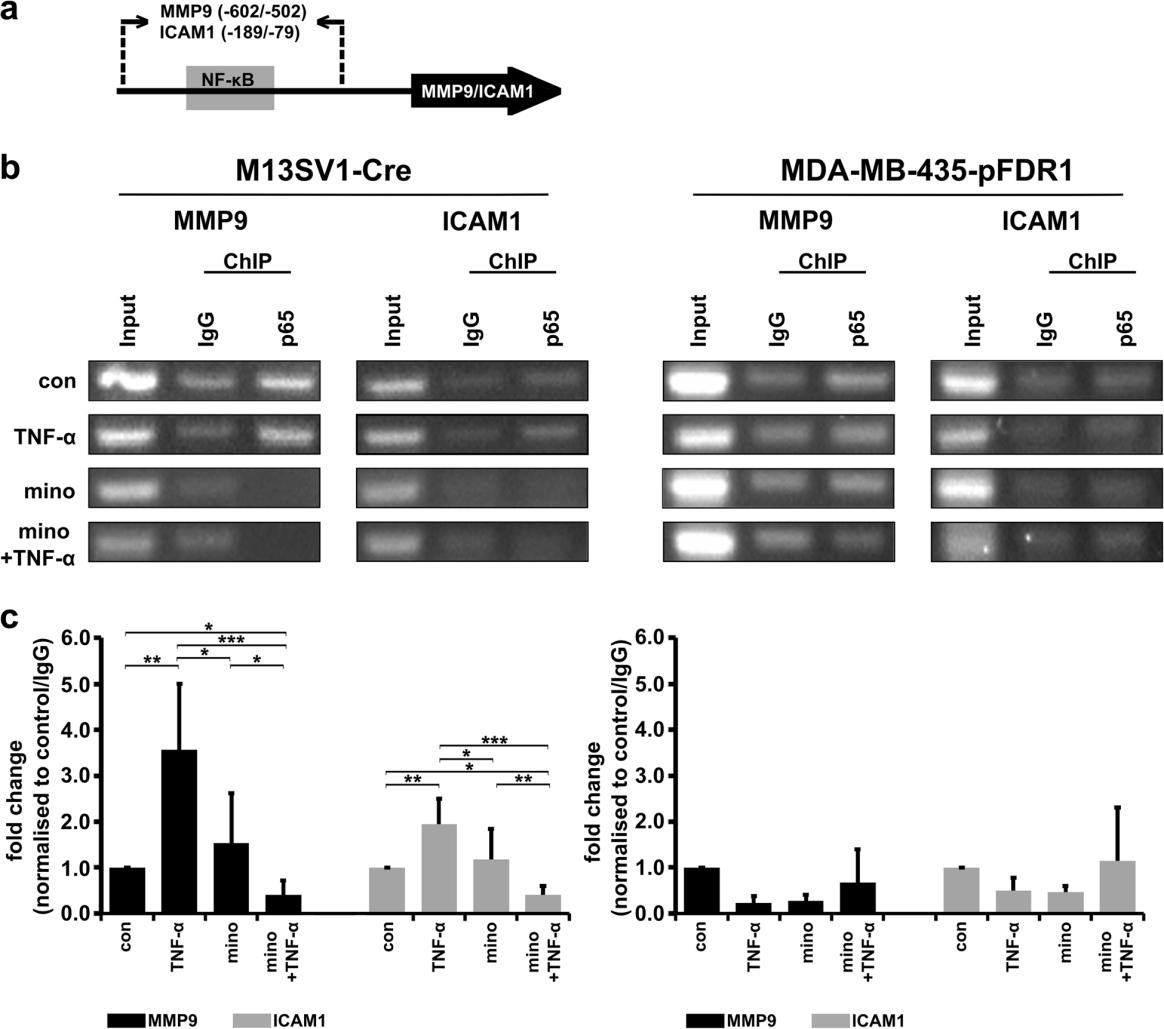
Minocycline suppresses TNF-α-induced NF-κB-dependent expression of MMP9 and ICAM1. NF-κB-binding activity on the promoter regions of ICAM1 and MMP9 was analysed by ChIP assay. **a** Schematic overview of the PCR and qPCR amplification strategy of MMP9 and ICAM1 promotor regions. **b** PCR data. **c** Validation of PCR data by qPCR. Representative data and the means ± STD of at least two independent experiments for MDA-MB-435-pFDR1 cells and three independent experiments for M13SV1-Cre cells are shown. Statistical analysis was conducted with the Kruskal-Wallis test: * = *p* < 0.05; ** = *p* < 0.01; *** = *p* < 0.001

Even though minocycline attenuates NF-κB activation similar to its nuclear translocation, we proceeded to clarify whether minocycline also disrupts NF-κB binding activity at the promoter regions of the MMP9 and ICAM1 genes. In a previous study, we demonstrated that cell fusion was positively triggered by TNF-α (100 ng/ml) and induced the expression of the proteinase MMP9 and the adhesion molecule ICAM1, which in turn was impaired by minocycline treatment [13]. We performed a chromatin immunoprecipitation (ChIP) assay using cells treated under the same conditions as described above. In fact, minocycline pre-treatment of M13SV1-Cre cells completely abrogated the TNF-α-induced NF-κB-p65 binding activity at MMP9 and ICAM1 promotor regions (Fig. 8), which is in agreement with our previously published data. However, in contrast to M13SV1-Cre cells, for MDA-MB-435-pFDR1 cells, the NF-κB-p65 yield was the highest in minocycline + TNF-α-treated cells, followed by untreated cells. Interestingly, the TNF-α-stimulated cells and the minocycline-treated MDA-MB-435-pFDR1 cells showed the lowest NF-κB binding activity (Fig. 8). In addition, even though TNF-α and minocycline led to NF-κB-p65 accumulation in the nucleus of MDA-MB-435-pFDR1 cells (Fig. 7a), ChIP data showed no TNF-α or minocycline promotor activity (Fig. 8). Nonetheless, the data confirmed that, in M13SV1-Cre cells, minocycline had an inhibitory effect by preventing the IKK/IκBα/NF-κB-induced gene expression of potential fusion factors, such as MMP9 and ICAM1.

### Minocycline inhibits TNF-α-induced activation of NF-κB-p65 gene expression

**Fig. 9:**
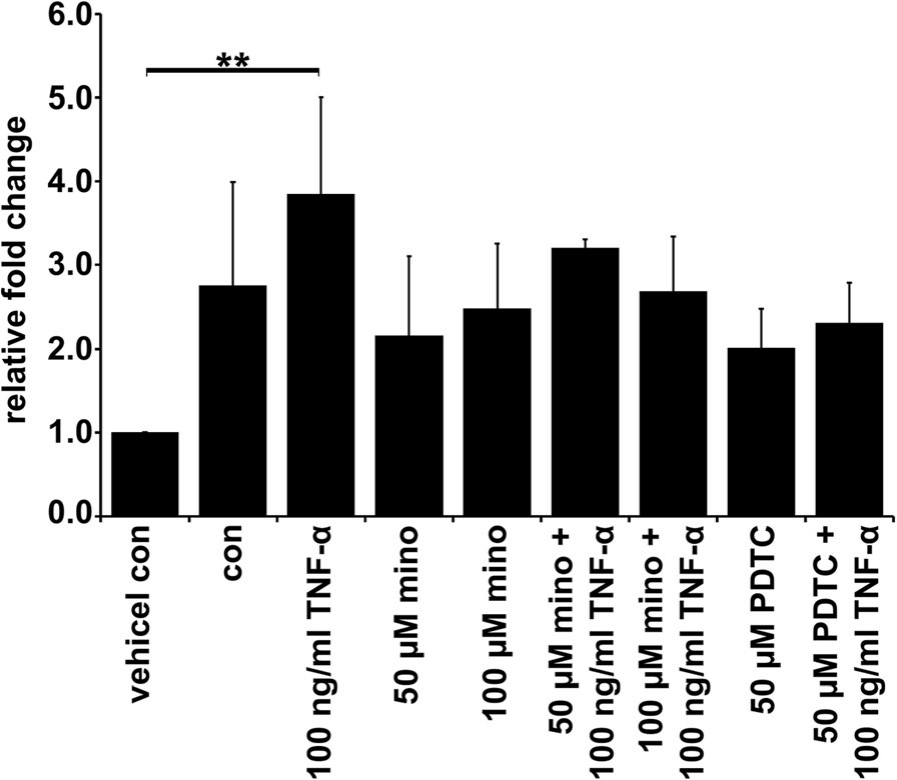
Minocycline inhibits TNF-α -induced activation of NF-κB-p65 gene expression in a dose-dependent manner. M13SV1-Cre cells were transiently transfected with an NF-κB promoter in a luciferase reporter vector and pre-incubated with different concentrations of minocycline (50 μM, 100 μM) or PDTC (50 μM) for 24 h, followed by TNF-α (100 ng/ml) stimulation for 2 h. Shown are the means ± STD from at least three independent experiments for which the luciferase activity of transfected cells was calculated in relation to nontransfected M13SV1-Cre cells, for which the value was set to 1. Statistical analysis was conducted with an ANOVA F-test and Scheffé post hoc test: * = *p* < 0.05; ** = *p* < 0.01; *** = *p* < 0.001

To further prove whether minocycline impairs NF-κB-mediated gene expression, an NF-κB-luciferase reporter assay was performed. Because the MDA-MB-435-pFDR1 breast cancer cell line was impossible to transfect with the NF-κB-luciferase reporter vector, no conclusion about NF-κB luciferin expression could be made for this cell line. The data revealed that minocycline inhibited TNF-α-induced NF-κB-dependent luciferase gene expression in a dose-dependent manner and was completely abolished with 100 μM minocycline in M13SV1-Cre cells (Fig. 9). As expected, no increase in TNF-α-induced NF-κB-luciferase activity could be detected in the PDTC-treated M13SV1 cells. In general, the luciferase activity was low in the PDTC-treated cell group than in the untreated control group. The results lead to the conclusion that minocycline is effective in preventing NF-κB-mediated gene expression in M13SV1-Cre cells as a consequence of its targeting of the NF-κB pathway, which suppresses its activation.

### Minocycline inhibits TNF-α-induced activation of NF-κB target-gene expression of protein products ICAM1 and MMP9

**Fig. 10:**
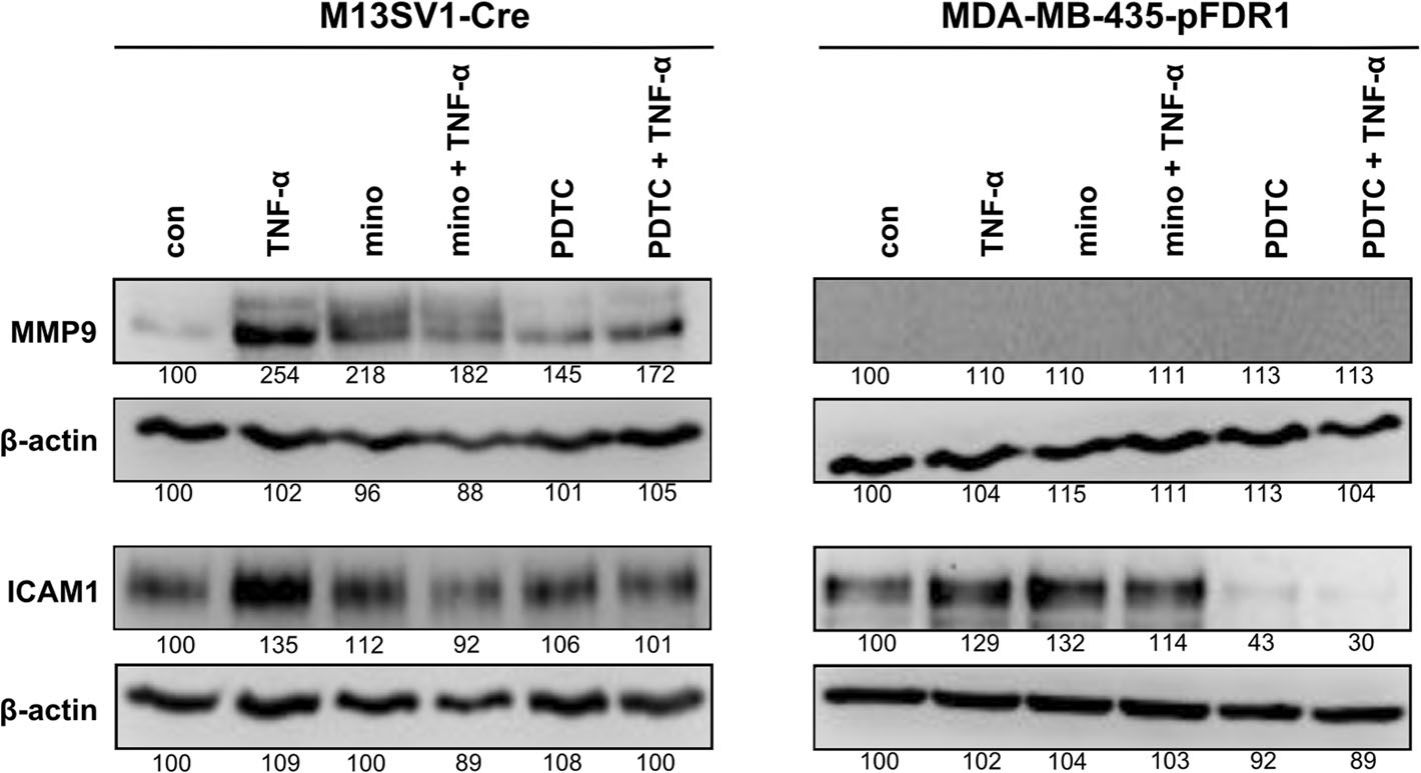
Minocycline impairs MMP9 and ICAM1 expression by inhibiting NF-κB activation. Cells were pre-incubated with minocycline (100 μM) or PDTC (50 μM) for 24 h and then subjected to TNF-α (100 ng/ml) stimulation for 2 h. Representative data from at least three independent experiments are shown

Finally, we investigated whether the protein expression of the putative cell fusion factors MMP9 and ICAM1 was managed by NF-κB activation. As described previously, minocycline treatment was successful in inhibiting MMP9 and ICAM1 protein targets [13]. Here, we used the NF-κB inhibitor PDTC as a control for the NF-κB-dependent expression of these targets. As expected, TNF-α induced MMP9 and ICAM1 expression levels were markedly decreased in PDTC treated M13SV1-Cre cells, which is accordance to minocycline data (Fig. 10) and previously published findings [13]. Likewise, no MMP9 expression was observed in MDA-MB-435-pFDR1 cells (Fig. 10), which is also in line with previously generated data [13]. Interestingly, PDTC treatment was much more effective in inhibiting basal and TNF-α induced ICAM1 expression in MDA-MB-435-pFDR1 cells than minocycline (Fig. 10). In any case, these data confirmed that minocycline interferes with NF-κB activity, thereby impairing the TNF-α induced expression of MMP9 and ICAM1 in M13SV1-Cre cells and ICAM1 in MDA-MB-435-pFDR1 cells.

## Discussion

In a previous study, we demonstrated that fusion between M13SV1-Cre and MDA-MB-435-pFDR1 cells could be induced by the pro-inflammatory cytokine TNF-α [9], a finding in agreement with inflammation as a positive trigger of cell fusion [46, 47]. It is generally accepted that inflammation in tumour tissue exists as a persistent condition and that inflammation processes directly affects the cells within and around the tumour [48, 49]. Several studies have reported that cell fusion was increased by inflammatory conditions, leading to a 10- to 100-fold higher cell fusion rate [50]. Nonetheless, less is known about the molecular mechanism and the underlying factors necessary for cell fusion between a tumour cell and a normal cell. In a recent study, we demonstrated that MMP9 is largely involved in the TNF-α-induced fusion of M13SV1-Cre and MDA-MB-435-pFDR1 cells, and this fusion was potently abolished by treatment with the tetracycline-based antibiotic minocycline [13]. Even though the effect of minocycline as an anti-inflammatory substance is well documented in numerous studies, the exact mechanism for this effect remains unknown. Minocycline may directly suppress iNOS, MMP9 or COX2 activity but might also interfere with the transcription factor NF-κB, thereby diminishing the inflammatory response [51]. Hence, the aim of this work was to clarify how minocycline impairs the TNF-α-induced fusion of M13SV1-Cre and MDA-MB-435-pFDR1 cells.

In the present study, the MDA-MB-435 cancer cell line was used, which is the subject controversy as it is debated whether it is of breast cancer or melanoma origin. Rae et al. showed that MDA-MB-435 cells and M14 melanoma cells exhibit a similar gene expression pattern and a comparative genomic hybridisation and microsatellite polymorphism profile [52, 53], suggesting that MDA-MB-435 cells are of melanoma origin. However, these cell lines exhibit different DNA hyper-methylation profiles [54, 55]. Likewise, MDA-MB-435 cancer cells possess properties that are indicative of breast cancer cells, such as those of MDA-MB-231, SUM1315 or HBL100, including the expression of β-casein, α-lactalbumin, epithelial membrane antigen (EMA), and keratin-19 [55]. Moreover, MDA-MB-435 cancer cells have the ability to produce and secrete milk lipids upon β-heregulin stimulation, which is a common marker for breast epithelial or breast cancer cells. These findings indicate that MDA-MB-435 cancer cells were of mammary origin.

Several studies have indicated a role for SRC activation in TNF-α signalling as a non-receptor tyrosine kinase bound to TNFR1, which modulates the inflammatory response by activation of the MAPK [38] or the AKT pathway [56]. However, western blot data revealed that TNF-α signalling was predominantly transduced in both cell lines via the TNFR1-TRAF2 axis but not via SRC. In M13SV1-Cre cells, SRC was not co-immunoprecipitated in TNF-α treated cells, and increased phospho-SRC levels not observed upon TNF-α stimulation. Similar findings were observed for MDA-MB-435-pFDR1 cells. However, weakly increased phospho-SRC levels were found in minocycline and TNF-α co-treated MDA-MB-435-pFDR1 cells, but we assume that this phosphorylation is attributable to the higher SRC expression levels concomitant with the basal phosphorylation observed in minocycline-treated cells and not due to a TNF-α-specific effect. Nonetheless, the frequency of the TNF-α-induced fusion of M13SV1-Cre and MDA-MB-435-pFDR1 cells was slightly decreased in a dose-dependent manner by the SRC inhibitor PP1, suggesting that a possible role for SRC in cell fusion could not be ruled out.

Several lines of evidence indicated that the JNK, ERK1/2 and p38 MAPKs could be activated by TNF-α via a TRAF2- and TGF-β–associated kinase 1 (TAK1)-dependent process [57–59]. For instance, TRAF2 knockdown has been associated with reduced p38 MAPK activation in macrophages [57]. Likewise, knockout of the death-domain kinase receptor-interacting protein (RIP) in mouse fibroblasts was also sufficient to decrease the activity of the ERK1/2, p38 and JNK MAPKs [59]. However, the role of MAPKs in the TNF-α-induced fusion of M13SV1-Cre cells and MDA-MB-435-pFDR1 cells remains to be clarified. Western blot data revealed that MAPKs were activated upon TNF-α stimulation in both cell lines and that minocycline had an impact on protein phosphorylation levels, which differed, however, between MAPK subtypes and the cell lines used. For instance, diminished phospho-ERK1/2 levels but markedly enhanced phospho-p38 levels were observed in minocycline-treated M13SV1-Cre cells. It is well recognised that minocycline could have a different impact on MAPK phosphorylation. For instance, slightly increased phospho-SRC and phospho-ERK1/2 levels were observed in CD3/CD28-stimulated CD4^+^ T cells that were treated for 24 h with 10 μM minocycline [60]. In contrast, Nikodemova et al. described an inhibitory effect of minocycline in blocking ERK1/2, p38 and JNK phosphorylation only after lipopolysaccharide (LPS) stimulation in microglia cells, but it had no effect on MAPK activation by H_2_O_2_ [61]. However, cell fusion studies revealed a marked increase in frequency of M13SV1-Cre and MDA-MB-435-pFDR1 cell fusion in the presence of specific MAPK inhibitors, suggesting a somewhat inhibitory role of MAPK signalling in the TNF-α-induced fusion of these cells. Conceivably, the pro-fusogenic effect of MAPK inhibition might also be attributed to impaired AP-1-induced gene expression. The transcription factor AP-1 is a main target of MAPK signalling and is involved in the TNF-α-induced expression of MMP9 [62]. However, data from Szeto et al. showed that minocycline did not alter the transcriptional activity of AP-1 in human CD4^+^ T cells [63]. In accordance with the increased cell fusion frequencies of M13SV1-Cre cells and MDA-MB-435-pFDR1 cells in the presence of MAPK inhibitors, a dose-dependent enhanced cell fusion rate was also observed for the specific AP-1 inhibitor SR11302. These findings indicate that MAPK signalling concomitant with AP-1 activation likely results in the engagement of an anti-fusogenic pathway or the expression of anti-fusogenic proteins. Hence, it would be of interest to investigate how the gene expression profile of M13SV1-Cre cells and MDA-MB-435-pFDR1 cells is altered by specific MAPK inhibitors and whether proteins exhibiting anti-fusogenic properties can be identified.

The anti-inflammatory effect of minocycline due to its targeting of NF-κB activity is well known and has been reported in several studies [24, 28, 60, 64]. However, it remains to be elucidated how exactly NF-κB activity is blocked by minocycline. Song et al. showed that NF-κB was downregulated in rat spinal astrocytes due to minocycline-dependent inhibition of IKKα phosphorylation, but IκBα phosphorylation levels remained unchanged [64]. Likewise, minocycline could target the NF-κB pathway at the TAK-TAB1 level, thereby suppressing IKK/IκBα activation and NF-κB-induced TGF-β1 expression [32]. Because IKKα/β levels remained unchanged in both cell lines and both TNF-α and minocycline led to slightly increased phospho-IKKα/β levels in M13SV1-Cre cells and MDA-MB-435-pFDR1 cells, we conclude that neither IKKα/β expression nor its activity was impaired by this antibiotic compound. Moreover, IκBα protein and phosphorylation levels were decreased only in M13SV1-Cre cells, indicating that minocycline may rather affect IκB signalling at the IκBα level. This would be in agreement with data from Naura and colleagues [60], who demonstrated that minocycline effectively blocked IκBα and NF-κB activation in CD4^+^ T cells in an IKKα/β-independent mechanism [60].

Interestingly, minocycline-mediated lower IκBα protein expression was not associated with increased nuclear levels of NF-κB in M13SV1-Cre cells. In contrast, overall lower NF-κB expression concomitant with decreased nuclear transport and NF-κB phosphorylation was found in minocycline- and minocycline + TNF-α-treated cells and correlated with reduced promoter activity as revealed by the promoter activity assay and the luciferase reporter assay. It cannot be ruled that the decreased nuclear transport and NF-κB phosphorylation were also to the results of a minocycline-specific effect, but we assume that, instead, this was caused by the decreased total NF-κB expression in minocycline-treated M13SV1-Cre cells. In any case, western blot data revealed that the expression of other proteins, such as TNFR1 and SRC, was diminished in minocycline-treated M13SV1-Cre cells. It is well known that TNF-α stimulation leads to the expression of NF-κB target genes, such as those for ICAM1 [41], MMP9 [40] and TRAF2 [65]. However, neither TNFR1 nor SRC belongs to the group of genes targeted by NF-κB, and it can therefore be concluded that minocycline impairs the overall gene expression rather than the NF-κB-specific gene expression of M13SV1-Cre cells. Moreover, compared to the results from the minocycline-only treatment, TNFR1, SRC, IκBα and NF-κB expression levels were diminished in M13SV1-Cre cells co-treated with minocycline + TNF-α. These data indicate that TNF-α likely fosters the inhibitory effect of minocycline on cell gene expression; however, it remains to be elucidated how TNF-α signalling interferes with minocycline activity.

In contrast to M13SV1-Cre cells expression levels of NF-κB, TNFR1 and SRC remained unchanged or even slightly increased in MDA-MB-435-pFDR1 cells treated with minocycline and minocycline + TNF-α, indicating that minocycline has a different impact on the gene expression pattern of diverse cell types. Moreover, increased nuclear levels of NF-κB were observed in MDA-MB-435-pFDR1 cells treated with minocycline and minocycline + TNF-α, suggesting that minocycline may act as an inducer of the NF-κB pathway in this cell line. Moreover, as shown here and in our previous study [13], ICAM1 expression levels were increased in minocycline + TNF-α-treated MDA-MB-435-pFDR1 cells. This finding is in agreement with data indicating that increased phosphorylation levels of NF-κB concomitant with increased expression of vascular cell adhesion molecule 1 (VCAM1) and intercellular adhesion molecule 1 (ICAM1) observed in minocycline-treated human mesenchymal stem cells (MSCs) [66, 67]. Even though MMP9 mRNA levels were also increased in MDA-MB-435-pFDR1 cells treated with minocycline and minocycline + TNF-α, only very low MMP9 protein expression could be determined [13]. ChIP assay data indicate enhanced binding of NF-κB to the promoter region of MMP9, which is in agreement with our previously published data revealing increased MMP9 mRNA levels in minocycline + TNF-α-treated MDA-MB-435-pFDR1 cells [13]. However, the regulation of NF-κB is complex and underlies a variety of process and modulation steps, as well as the interaction of diverse enhancers/repressors and other factors, that interfere with the NF-κB signal network to regulate or dysregulate its activation [45]. It is possible that, in MDA-MB-435-pFDR1 cells, the NF-κB p65 subunit binds to different κB sites within the promoter regions of the MMP9 gene that were not targeted by the primers used. Likewise, it was reported that IκBα could enter the nucleus and bind to NF-κB, thereby abrogating transcriptional activity [68]. As a consequence, nuclear-translocated NF-κB was detected but was found to be inactive. Furthermore, transcriptional activity could also be impaired by a time-limited degradation of the transcription activity itself [45]. We consider these possibilities to unlikely since the qPCR data clearly showed increased MMP9 mRNA expression in minocycline + TNF-α-treated MDA-MB-435-pFDR1 cells. Thus, instead, we conclude that pre- or post-transcriptional processes might be responsible for the very low MMP9 protein expression levels in MDA-MB-435-pFDR1 cells.

**Fig. 11:**
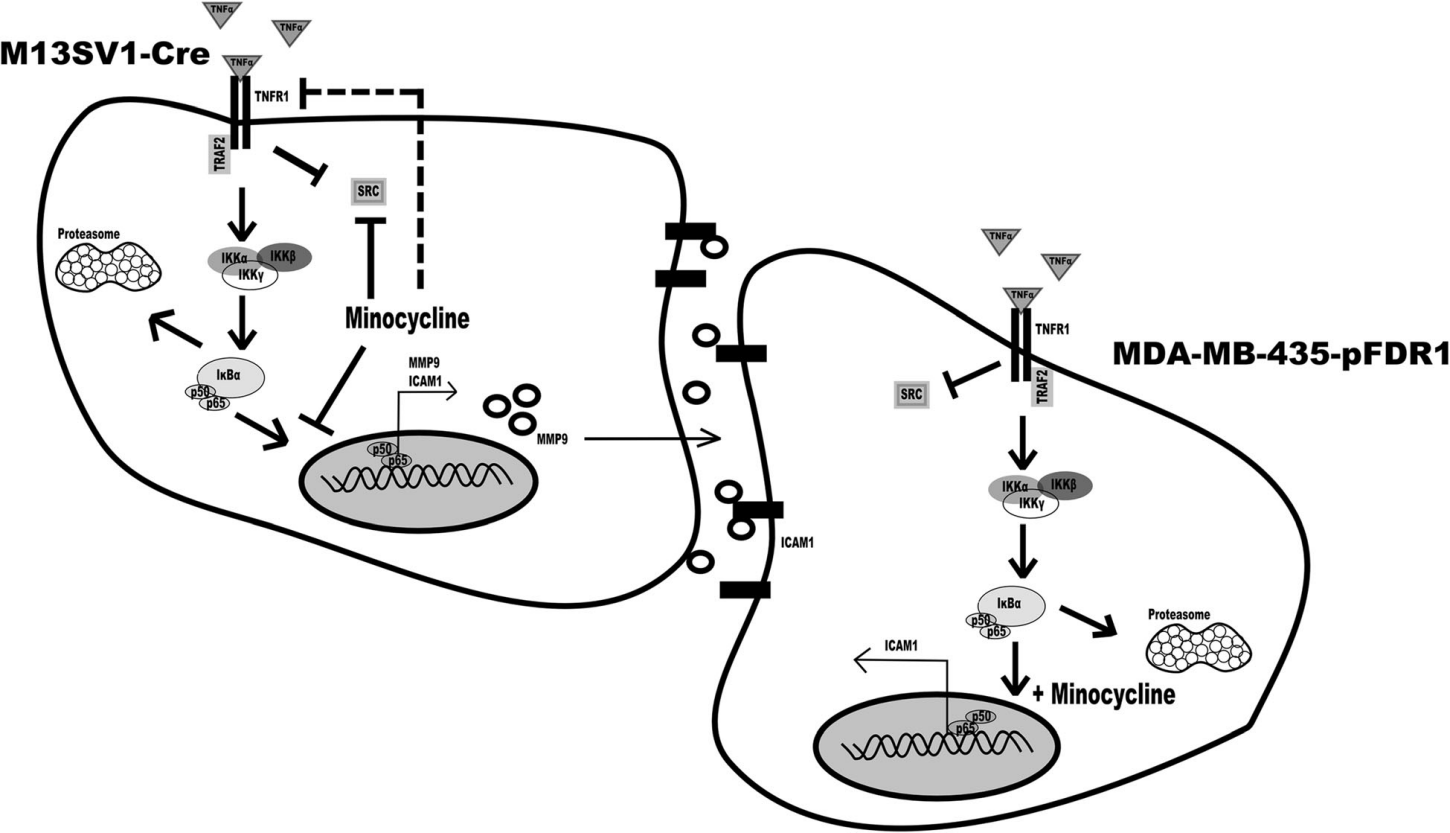
Schematic model of how minocycline may inhibit TNF-α-induced cell fusion. The intracellular signal transduction pathway by TNF-α was investigated in this study. When TNF-α binds to TNFR1, the downstream signal cascade is activated via TRAF2 recruitment and IκBα kinase complexes resulting in the release of the transcription factor NF-κB in M13SV1-Cre cells. Conjointly, the TNFR1-SRC interaction is abrogated during the signal processing of TNFR1-TRAF2. Minocycline inhibits TNFR1/TRAF2/IKK/IκBα/NF-κB signalling activity in M13SV1 cells, thereby suppressing the NF-κB-regulated target gene expression of putative cell fusion factors, such as MMP9 and ICAM1. Furthermore, SRC and TNFR1 expression was also suppressed by minocycline in M13SV1-Cre cells, which may additionally contribute to an impaired TNF-α induced TNFR1-NF-κB signalling. In contrast to M13SV1-Cre cells, minocycline in combination with TNF-α induces high NF-κB-regulated ICAM1 expression in MDA-MB-435-pFDR1 cancer cells. It is suggested that the release of MMP9 is responsible for the ICAM1 surface shedding that reduces the distance of the fusion partners, thus facilitating membrane merging and the initiation of the fusion process

In any case, our data strongly suggest the necessity of NF-κB activity for TNF-α induction of M13SV1-Cre and MDA-MB-435-pFDR1 cell fusion. This evidence was further supported by cell fusion studies using the NF-κB inhibitor PDTC, which blocked the TNF-α-induced fusion of the cells in a dose-dependent manner. Likewise, treatment of the cells with PDTC was correlated with decreased MMP9 expression in M13SV1-Cre cells and decreased ICAM1 expression in both cell lines, further indicating that both MMP9 and ICAM1 were NF-κB target genes. Our data also showed that both cell lines responded differently to minocycline, suggesting that minocycline may act as a double-edged sword. On the one hand, minocycline was a potent inhibitor of (NF-κB-dependent) gene expression in M13SV1-Cre cells, whereas in MDA-MB-435-pFDR1 cells, TNF-α signalling concomitant with NF-κB activity was enhanced (Fig. 11). However, how does minocycline impair protein expression in M13SV1-Cre cells? MMP9, ICAM1, IκBα and NF-κB are all NF-κB target genes, which aligns with findings showing that minocycline targets the NF-κB pathway [51]. Inhibition of NF-κB signalling by minocycline is correlated with lower expression levels of NF-κB target genes. However, TNFR1 and SRC do not belong to the group of NF-κB target genes, but decreased expression levels of these proteins were observed in minocycline-treated M13SV1-Cre cells. Thus, it might be assumed that in addition to NF-κB signalling and NF-κB-mediated gene expression, other signal transduction pathways and transcription factors might be affected by minocycline; this possibility should be investigated in ongoing studies. Likewise, it remains to be explained why co-treatment of M13SV1-Cre cells with minocycline + TNF-α was more effective in inhibiting protein expression than minocycline alone. The query also concerns the observation of markedly enhanced nuclear NF-κB levels in minocycline + TNF-α co-treated MDA-MB-435-pFDR1 cells compared to cells treated with minocycline only or TNF-α.

## Conclusion

In summary, we conclude from our data that targeting the TNFR1/TRAF2/IKK/NFκB signal transduction pathway might be a strategy for inhibiting inflammation-related cell fusion events within a tumour as it could give rise to cancer cell ×normal cell hybrids that could exhibit novel properties. Whether minocycline or other inhibitors in the NF-κB signalling pathway might be suitable compounds for anticancer treatment has to be determined in ongoing studies.

## Data Availability

The data sets used and/or analysed during the current study are available from the corresponding author upon reasonable request.

## Abbreviations

ADAM10: A disintegrin and metalloproteinase domain-containing protein 10
ADAM12: A disintegrin and metalloproteinase domain-containing protein 12
AP-1: Activator protein 1
ChIP: chromatin immunoprecipitation
COX-2: Cyclooxygenase-2
DMEM: Dulbecco’s minimal essential medium
EGF: Epidermal growth factor
EMA: Epithelial membrane antigen
ERK1/2: Extracellular signal-regulated kinases
ESA: Epithelial specific antigen
FCS: Foetal calf serum
FDR: Fluorescence double reporter
ICAM1: Intercellular cell adhesion molecule 1
IKK: IκB kinase
IL-13: Interleukin-13
IL-4: Interleukin-4
iNOS: inducible nitric oxide synthase
IκBα: Inhibitor of kappa B protein NF-κB nuclear factor kappa B
JNK: c-Jun NH2-terminal kinase
MAPK: Mitogen-activated protein kinase
M-CSF: Macrophage-colony stimulating factor
MMP9: Matrix metalloproteinase 9
MSC: Mesenchymal stem cells
MT1-MMP: Membrane-type matrix metalloproteinase 1
NFAT: Nuclear factor of activated T cells
NFATC2: Nuclear factor of activated T cells, cytoplasmic 2
NF-κB: Nuclear factor-κB
PBS: Phosphate-buffered saline
PCR: Polymerase chain reaction
qPCR: quantitative polymerase chain reaction
RAC1: Ras-related C3 botulinum toxin substrate 1
RANKL: receptor activator of nuclear factor kappa-B ligand
SDS-PAGE: sodium dodecyl sulphate-polyacrylamide gel electrophoresis
siRNA: small interfering RNA
STAT-6: Signal transducer and activator of transcription 6
TNFR1: Tumour necrosis factor receptor 1
TNF-α: tumour necrosis factor-α
TRAF2: TNF receptor-associated factor 2
VCAM-1: Vascular cell adhesion protein 1
Wnt/β-catenin: Wingless/Integrated β-catenin
